# A cellular census of human peripheral immune cells identifies novel cell states in lung diseases

**DOI:** 10.1002/ctm2.579

**Published:** 2021-11-24

**Authors:** Dongli Song, Furong Yan, Huirong Fu, Liyang Li, Jie Hao, Zhenhua Zhu, Ling Ye, Yong Zhang, Meiling Jin, Lihua Dai, Hao Fang, Zhenju Song, Duojiao Wu, Xiangdong Wang

**Affiliations:** ^1^ Zhongshan Hospital Department of Pulmonary and Critical Care Medicine Institute for Clinical Science Shanghai Medical University Fudan University Shanghai China; ^2^ Department of Emergency Zhongshan Hospital Shanghai Medical University Fudan University Shanghai China; ^3^ Shanghai Institute of Clinical Bioinformatics Shanghai Engineering Research for AI Technology for Cardiopulmonary Diseases Shanghai China; ^4^ Jinshan Hospital Centre for Tumour Diagnosis and Therapy Shanghai Medical University Fudan University Shanghai China; ^5^ Department of Emergency Shidong Hospital of Yangpu District Shanghai China; ^6^ Department of Anesthesiology Zhongshan Hospital Shanghai Medical University Fudan University Shanghai China

**Keywords:** acute lung pneumonia, immunity, lung diseases, PD‐1

## Abstract

Increasing evidence supports a central role of the immune system in lung diseases. Understanding how immunological alterations between lung diseases provide opportunities for immunotherapy. Exhausted T cells play a key role of immune suppression in lung cancer and chronic obstructive pulmonary disease was proved in our previous study. The present study aims to furthermore define molecular landscapes and heterogeneity of systemic immune cell target proteomic and transcriptomic profiles and interactions between circulating immune cells and lung residential cells in various lung diseases. We firstly measured target proteomic profiles of circulating immune cells from healthy volunteers and patients with stable pneumonia, stable asthma, acute asthma, acute exacerbation of chronic obstructive pulmonary disease, chronic obstructive pulmonary disease and lung cancer, using single‐cell analysis by cytometry by time‐of‐flight with 42 antibodies. The nine immune cells landscape was mapped among those respiratory system diseases, including CD4^+^ T cells, CD8^+^ T cells, dendritic cells, B cells, eosinophil, γδT cells, monocytes, neutrophil and natural killer cells. The double‐negative T cells and exhausted CD4^+^ central memory T cells subset were identified in patients with acute pneumonia. This T subset expressed higher levels of T‐cell immunoglobulin and mucin domain‐containing protein 3 (Tim3) and T‐cell immunoreceptor with Ig and ITIM domains (TIGIT) in patients with acute pneumonia and stable pneumonia. Biological processes and pathways of immune cells including immune response activation, regulation of cell cycle and pathways in cancer in peripheral blood immune cells were defined by bulk RNA sequencing (RNA‐seq). The heterogeneity among immune cells including CD4^+^, CD8^+^ T cells and NK T cells by single immune cell RNA‐seq with significant difference was found by single‐cell sequencing. The effect of interstitial telocytes on the immune cell types and immune function was finally studied and the expressions of CD8a and chemokine C–C motif receptor 7 (CCR7) were increased significantly in co‐cultured groups. Our data indicate that proteomic and transcriptomic profiles and heterogeneity of circulating immune cells provides new insights for understanding new molecular mechanisms of immune cell function, interaction and modulation as a source to identify and develop biomarkers and targets for lung diseases.

## Graphical abstract

1. New clusters and functions of circulating immune cells can be an important source to understand molecular mechanisms, identify disease‐specific biomarkers, and develop new target therapies.

2. The difference of biological processes and pathways of PBICs by single‐cell sequencing and RNAseq varies among diseases.

3. The interstitial cells could alter the differentiation and development of the immune cell types and immune function.

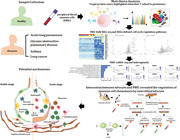

## 1 INTRODUCTION

Pneumonia, chronic obstructive pulmonary disease (COPD), asthma and lung cancer (LC) are most common and critical pulmonary diseases, accounting for majority of diseases in the respiratory system. Of multiple involvements, the immune system plays a central role in the initiation, exacerbation, development and prognosis of lung diseases. Dynamics and severities of lung diseases are highly dependent upon the exposure of antigens, patient predispositions and types of immune response.^1^, ^2^, ^3^ The dysregulation of immune cells in lung diseases is characterized by inflammatory cellular infiltration into the lung, resulting in progressive lung inflammation, tissue injury and cell remodelling.

In response to sustained antigen activation or chronic infection, T cells transit into exhaustion state characterized by overexpressed immune checkpoints and impaired effector function. Exhausted CD4^+^ or CD8^+^ T cells (Tex cells) develop during the progression of cancer and other chronic infections and are characterized by co‐express multiple inhibitory receptors, including T‐cell immunoreceptor with Ig and ITIM domains (TIGIT) and programmed cell death protein‐1 (PD‐1).^4^ Alterations of Tex cell transcriptional, epigenetic, metabolic and differential programmes play different roles in determining outcome in diseases.^5^ Tex cell‐targeted manipulations lead to new therapeutic opportunities for lung diseases. The application of antibodies against PD‐1 shows clinical therapeutic benefits for cancer therapy and potentials for infectious disease.^6^ Our previous studies defined that in patients with acute exacerbation of chronic obstructive pulmonary disease (AECOPD), the metabolic insufficiency of exhausted CD4^+^ or CD8^+^ T cells occurred and the targeting glycolysis of T cells restored immune functions.^7^ However, the full landscapes and atlas of circulating immune cell clusters and multi‐omic profiles between LC and noncancer diseases, between acute and chronic lung diseases, and between acute lung primary infection and stable station are unclear.

Alternations of Tex cells characteristics are considered as a new source to identify disease‐specific biomarkers in different disease settings, during which progenitor and more terminally exhausted populations of Tex cells were identified.^8^ Memory T cells, including central memory T cells (TCM) and effector memory T cells (TEM), could rapidly produce proinflammatory mediators in response to pathogens and were involved in pre‐existing immune memory acquired from previous infection with seasonal coronaviruses.^9^, ^10^ TCM and TEM recirculate between the blood and lymphoid organs or peripheral tissues, respectively.^11^ However, it is still unclear whether there are differences among Tex subsets or new clusters in memory T cells in lung diseases, especially between disease types. Double‐negative T (DNT) cells as one of T‐cell subsets are poorly understood, although DNT cells have the expendability and key roles in maintenance of autoimmune conditions in patients with systemic lupus erythematosus and psoriasis.^12^ DNT cells infiltrated into target organs and produced proinflammatory mediators, such as interferon γ (IFN‐γ) and IL‐17, in diseases.^13^

Systematic analysis of disease‐specific immunological signatures in patients is an important approach to understand molecular mechanisms and uncover new biomarkers.^14^ The aims of the present study are to map molecular landscapes, identify possible signal pathways by analyzing heterogeneity of circulating immune cell target proteomic and transcriptomic profiles, and try to intervene the function of immune cells by the interactions between circulating immune cells and lung residential cells in various lung diseases. We first measured 42 target penal profiles of peripheral blood immune cells (PBIC) from healthy volunteers and patients with acute pneumonia, stable pneumonia, stable asthma, acute asthma, COPD, AECOPD, and LC using single‐cell analysis of cytometry by time‐of‐flight (CyTOF). We specially focused on the differences of PBIC proportions and distribution between LC and noncancer diseases, between acute and chronic lung diseases, and between acute lung primary infection and secondary infection. To explore potential molecular mechanisms, biological processes and pathways with significant differences were investigated using PBIC bulk RNA‐seq, and the heterogeneity and interactions among PBICs were defined by single‐cell RNA‐sequencing (scRNA‐seq). To furthermore understand the interaction between lung resident cells and PBICs, we selected lung telocytes (TCs) as the representative of lung interstitial cells to study effect of interstitial cells on the immune cell types and immune functions.

## 2 METHODS

### 2.1 Measurement of target proteomic profiles using CyTOF

The current study was approved by the Fudan University Ethical Committee for human experiments (B2019‐197). Healthy volunteers of age from 39 to 50 years old, two females and three males, did not take over the counter supplement or were not sick with some common illnesses such as a common cold and claimed to be without immune diseases. Six patients diagnosed with acute asthma or stable asthma, of age from 33 to 56 years old, including three females and three males. Patients with acute pneumonia, stable pneumonia, AECOPD, COPD or LC patients were recruited from departments of emergency, respiratory medicine and critical care medicine.^7^ Of patients with pneumonia, one suffered from bacterial and mycoplasma infection, two from fungus infection, two from bacterial infection alone, and two from unknown pathogen. Peripheral blood was collected from patients with acute pneumonia, stable pneumonia, stable asthma, acute asthma, COPD, AECOPD or LC before receiving therapy during the hospitalization, while the samples of stable pneumonia patients were collected and turned to stable condition after receiving antibiotic treatment. Clinical phenome characteristics of patients with acute pneumonia, stable pneumonia, COPD, AECOPD or LC for immunophenotyping are listed in Table 1 and stages of patients with LC are shown in Table S2. Peripheral blood was isolated from patients or healthy individuals and collected and stored in ethylene diamine tetraacetic acid (EDTA) tubes. All samples were delivered at 4°C and were processed immediately. PBICs were stained for flow cytometric sample preparation according to the protocol described in the CyTOF section.

**TABLE 1:** Clinical phenome characteristics of patients with acute pneumonia, stable pneumonia, AECOPD, COPD or LC for immunophenotyping (mean±SD)

Characteristics	acute pneumonia	stable pneumonia	AECOPD	COPD	LC
Number	n = 6	n = 3	n = 5	n = 5	n = 5
Age	53.43 ± 20.59	42.50 ± 12.82	70.40 ± 8.76	73.80 ± 9.01	56.00 ± 15.08
Sex					
Male	5	2	5	5	5
Female	2	2	0	0	0
Pack‐years of smoking mean			46 ± 13.42	45 ± 22.91	20.00 ± 34.64
Current smokers			0	2	1
Ex‐smokers			5	3	1
History of COPD(Years)			13.20 ± 12.78	14 ± 5.48	0
BMI kg/m^2^					20.86 ± 3.60
weight					61.40 ± 9.48
height					1.72 ± 0.05
C‐reactive protein (mg/L^‐1^)	129.33 ± 121.04	169.00 ± 198.10	50.92 ± 42.18	53.74 ± 23.75	72.00 ± 83.21
Oxygen partial pressure(mmHg)	87.76 ± 31.85		74.21 ± 17.68	100.31 ± 36.87	
Carbon dioxide(mmHg)	21.43 ± 3.99		42.64 ± 8.15	45.32 ± 17.32	27.00 ± 1.41
Leukocyte(*10^^^9/L)	1.07 ± 0.65	1.53 ± 0.56	6.00 ± 2.41	9.53 ± 2.45	6.68 ± 1.27
Monocyte percentage (%)	7.80 ± 3.89	9.88 ± 5.05	8.10 ± 3.64	7.57 ± 2.02	11.26 ± 4.22
Neutrophil(*10^^^9/L)	7.56 ± 4.00	4.05 ± 1.76	17.51 ± 29.33	34.18 ± 37.69	4.70 ± 1.79
Monocyte(*10^^^9/L)	0.64 ± 0.29	0.57 ± 0.72	0.55 ± 0.16	0.65 ± 0.19	0.73 ± 0.22

### 2.2 Cell suspension for mass cytometry

Blood samples were first depleted of the red blood cells using cells lysis buffer. Cells were then washed using ice‐cold physiological buffer solution (PBS) and were centrifugalized to remove red blood lysis. Cells were resuspended and filtered using 40‐μm filter (BD Bioscience, NJ, USA). Cell suspensions were then diluted to final concentration of 3 × 10^6^ cells per 100 μl buffer.

### 2.3 Antibodies for mass cytometry

Antibodies with metal‐conjugated as available targets were obtained from the companies listed in Table S2. These antibodies were prepared following the manufacturer's instructions (Fluidigm, South San Francisco, CA). Maxpar Antibody Labeling Kit was used to prepare antibody, conjugated and preserved in antibody stabilization buffer (Candor Biosciences, Constance, Germany) containing PBS and 0.05% NaN_3_. Titration was performed with all antibodies before staining.

### 2.4 Mass cytometry

Cells were washed in ice‐cold PBS and stained with 5 mM cisplatin (Fluidigm) for viability stain in order to distinguish living cells from dead cells. Antibodies were added into the cell dilution and placed in ice‐cold flow cytometry (FACS) buffer containing 1.25% bovine serum albumin in PBS (BD Bioscience) for Fc receptor blocking. The cells were incubated with the antibody in ice‐cold buffer for 1 h for staining.

Cells were washed with pre‐cooled FACS buffer and Milli‐Q water (Heal Force Smart N, Shanghai, China). After being resuspended with Milli‐Q water, cells were filtered with 35‐μm filters (Falcon, BD Bioscience). EQ four element calibration beads (Fluidigm) diluted by 1:10 were used as loading control. Cells were examined with a Helios3 CyTOF mass cytometer (Fluidigm). Maximum sample loading rate was 300 events per second. Then, the data files were standardized and analyzed using the bead‐based normalizer^15^ and cell analysis bank (https://www.cytobank.org/), respectively. CD45^+^ T cell was analyzed with the average cell number of 237,585.5 per sample and that of CD3^+^ T cell was 31,632.3. The next analysis including data of healthy, acute pneumonia, stable pneumonia, acute asthma, stable asthma, COPD, AECOPD and LC were analyzed as reported previously.^7^ t‐Distributed stochastic neighbour embedding (t‐SNE) dimension reduction was performed using R package. The clustering of PhenoGraph map and the dimensionality reduction were completed using R software package cytofkit and the default *k* value = 30.^16^, ^17^ According to the median of each phenological group marker expression, two meta clusters were determined by hierarchical clustering method.^17^ Data were stored in a public repository (access link: https://202.108.211.75).

### 2.5 Single‐cell RNA sequencing

For the measurement of scRNA‐seq, isolated cells were counted and diluted to the final concentration within 1200 cells per microlitre with a minimum cell viability of 80%. Single cells were isolated on a chromium controller (BD plateform, BD Bioscience) following instructions from the manufacturer and as reported previously.^7^ Single cells were captured using BD Rhapsody Single‐Cell Analysis System with the BD Human Sample Multiplexing Kit, BD Rhapsody Cartridge Kit, BD Rhapsody Cartridge Reagent Kit and BD Rhapsody cDNA Kit (BD Bioscience). After cDNA synthesis, RNA‐sequencing libraries were performed using the BD Rhapsody WTA Amplification Kit according to the manufacturer's instructions (BD Bioscience). Libraries were sequenced using a NextSeq 500/550 v2.5 High‐Output Kit and an Illumina NextSeq (Illumina, San Diego, CA, USA). Data were stored in a public repository (access link: https://202.108.211.75).

### 2.6 Isolation and identification for primary TCs

Primary human lung TCs were isolated and stained with immunofluorescence for primary TCs identification as reported previously.^18^, ^19^ TCs were incubated on the slides with primary monoclonal antibodies against vimentin (Abcam, CA, USA), platelet‐derived growth factor (Cell Signaling Technology, Whitby, ON, Canada) and forkhead box L1 (Novus Biologicals, CO, USA) after fixing and blocking. Those antibodies were incubated overnight at a 1:100 dilution. The slides were then incubated with corresponding secondary antibodies diluted at 1:100 in blocking buffer for 2 h in dark. Then cell nucleus was stained with DAPI at 1:200 dilution (Sigma Aldrich, Merck, Beijing, China). The slides were evaluated with a Leica DM4000 B fluorescence microscope.

### 2.7 Cell co‐culture

Co‐culture of TCs and immune cells was performed as direct co‐culture or Transwell co‐culture, respectively. In the direct co‐culture system, 5 × 10^4^ TCs and immune cells were mixed and plated on 24‐well plates for 24 h. In the Transwell co‐culture system, 5 × 10^4^ immune cells were plated at the bottom of the well and 5 × 10^4^ TCs cells were plated at the upper chamber above the Transwell membrane with 0.4‐μm pore size (Corning, Merck) for 24 h. The experiment was separated into three groups: control group, lipopolysaccharide (LPS)‐induced TCs group and LPS‐induced both cell types group. The final LPS concentration in the LPS‐induced group was 1 μg/ml for 24 h.

### 2.8 PBIC bulk RNA‐seq and scRNA‐seq data analysis and mining

The data were imported and quality control and downstream analysis were performed using the Seurat (v3) R toolkit. Unless otherwise specified, function genes were run with default parameters. The datasets from different samples were integrated to remove the batch effect using Seurat v3 with default parameters. The highly variable genes were identified by the Seurat function ‘FindVariableFeatures’. The top 2000 highly variable genes were used for data integration. ‘ScaleData’ was applied to scale the data. ‘FindNeighbors’ and ‘FindClusters’ functions were used for the first 20 principal components adopted for autoclustering analyses. UMAP scatter plot was used for visualizing the clustering results. The different expression genes (DEGs) were obtained by comparing between control versus LC, control versus AECOPD, and LC versus AECOPD, respectively, using *t*‐test. The genes with false discovery rate <0.05 and log2 fold change >1 were identified as DEGs. In the volcano plot and heatmap, only the DEGs with log2 fold change >2 were shown. Differentially expressed genes between groups were identified by biological process analysis using GSEA (http://software.broadinstitute.org/gsea/msigdb/annotate.jsp) and KEGG enrichment, respectively. Data were stored in a public repository with access link: https://202.108.211.75.

### 2.9 Statistics

Wilcoxon rank‐sum test was used for statistical significance. After the analysis of nonparametric one‐way analysis of variance (ANOVA) for multiple groups, the Student's *t*‐test was applied for comparisons of DEGs between two groups. The genes with false discovery rate <0.05 and log2 fold change >1 were identified as DEGs. *p*‐Values less than .05 are considered as statistically significant.

## 3 RESULT

### 3.1 Target proteomic profiles of PBICs among lung diseases

**FIGURE 1:**
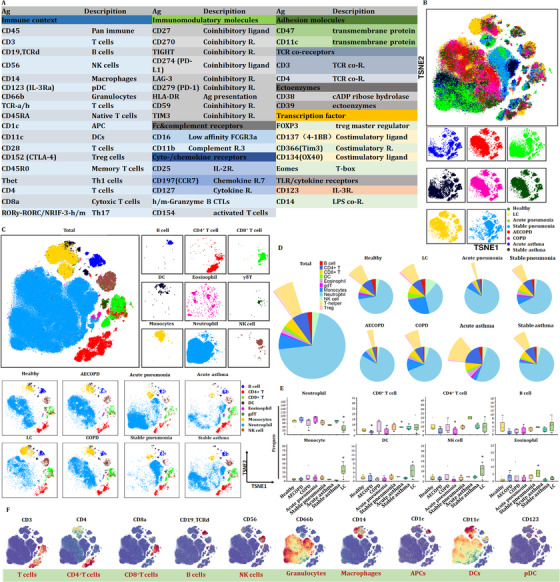
Target protein omic for the immune composition in healthy group and patients. (A) The panel of the CyTOF process. CyTOF analysis at single‐cell level was used to perform target protein omics and explore the immune composition of healthy volunteers and patients with lung diseases. (B) t‐SNE maps displaying 100 000 cells from the PBMC analyzed with immune cell subsets in healthy controls, patients with acute pneumonia, stable pneumonia, stable asthma, acute asthma, COPD, AECOPD and lung cancer (LC). (C) Distribution of immune subsets in volunteers. The distributions of B cells, CD4^+^ T cells, CD8^+^ T cells, DC, eosinophils, γδT, monocytes, neutrophils and NK cells were analyzed in all volunteers and in healthy controls and patients. (D) Pie chart displaying the proportion of immune cell subsets in the peripheral blood from healthy volunteers and patients with lung disease. CD4^+^ T cells and CD8^+^ T cells showed alterations in healthy controls and patients with acute pneumonia, AECOPD and acute asthma. (E) Bar plots of D. Frequencies of B cells, CD4^+^ T cells, CD8^+^ T cells, DC, eosinophils, γδT, monocytes, neutrophils and NK cells in healthy controls and patients. (F) Markers analyzed with immune cell subsets. The distributions of CD3, CD4, CD8a and others were analyzed and shown in all volunteers

We first estimated the basic immune status and compared the differentiations in PBIC of patients with lung diseases and healthy subjects. The difference of relative frequencies of cell types and molecular phenotypes was detected with CyTOF. Approximately 4 × 10^6^ single cells from the peripheral blood of healthy volunteers and patients with acute pneumonia, stable pneumonia, acute asthma, stable asthma, COPD, AECOPD or LC were analyzed (Figure 1A). Figure 1B,C reveals the distribution and frequency of cells, including CD4^+^ T cells, CD8^+^ T cells, γδT, DNT, monocytes, dendritic cells (DC), B cells, natural killer cells, eosinophils and neutrophils. Clusters were biologically annotated based on the relative abundance of markers expression. The proportion and frequency of CD4^+^ T cells were significantly decreased while that of CD8^+^ T cells were significantly increased in patients with acute pneumonia. The proportion and frequency of monocyte and NK cells were significantly increased in patients with LC. The proportion and frequency of eosinophils were significantly increased in patients with acute asthma (Figure 1D,E). Similar cell subsets were shown in our results and CD8^+^ T cells showed an obvious proportion advantage beyond neutrophils. Furthermore, CD8^+^ T cells were significantly decreased in patients with AECOPD or acute pneumonia. Analysis with signature markers (e.g., CD3, CD4 and CD8a) identified the distribution of cell subsets (e.g., T cells, CD4^+^ T cells and CD8^+^ T cells) (Figure 1F).

**FIGURE 2:**
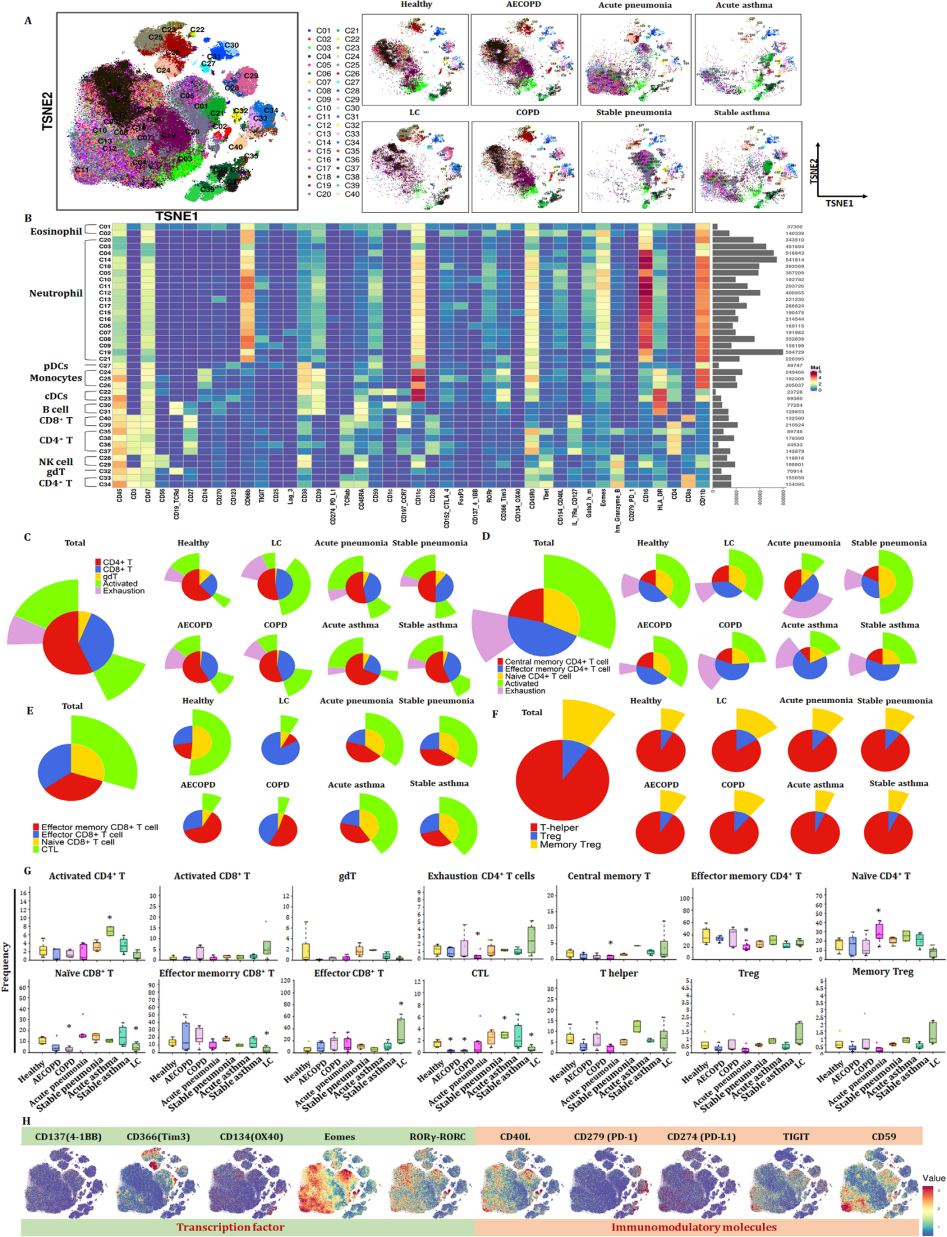
Identification of the peripheral immune cell populations defined Tex in pneumonia. (A) t‐SNE maps displaying 100 000 cells from the PBMC analyzed with immune cell subsets in healthy controls and patients with lung diseases. We analyzed 40 clusters within immune cells and showed the difference in the distribution between healthy controls and patients with lung diseases. (B) Heatmap of immune subsets in healthy controls and patients with lung diseases. Clustering analysis for markers distinguishing 19 neutrophil subsets, six CD4^+^ T cells subsets, two CD8^+^ T‐cell subsets, pDCs, two cDC subsets, three monocytes and two B‐cell subsets. The markers expression on these subsets is shown in Table 2. (C–F) Pie chart displaying the frequencies of immune cell subsets in the peripheral T cells from healthy volunteers and patients with lung disease. Ratios of exhausted cells were analyzed (C). CD4^+^ exhausted T cells were analyzed (D). CD8^+^ T‐cell subsets were analyzed (E). T‐helper cells, regulatory T cells (Treg) and memory Treg were analyzed (F). (G) Bar plots of (C)–(F). The frequencies of CD4^+^ T‐cell subsets and CD8^+^ T‐cell subsets were analyzed. (H) Markers analyzed with immune molecules. The distribution of transcription factors and immunomodulatory molecules in all samples are displayed

We further identified clusters and distributions of approximately 40‐cell subsets in healthy volunteers and patients with lung diseases (Figure 2A). Two clusters in eosinophil, cDCs, B cell and NK cell, three clusters of monocytes and pDCs were identified. T cells were analyzed with eight clusters including five CD4^+^ T cells, two CD8^+^ T cells and γδT. The distribution of clusters was more complex than that of subsets (Figure 1B,C). To obtain more robust interpretations, clusters were defined by marker set signatures rather than single markers, and based cluster annotation on the expression of canonical marker signatures (e.g., eosinophils, neutrophils, pDCs, monocytes, cDCs, B cells, CD8^+^ T, CD4^+^ T, natural killer cells and γδT). The generalizability of all clusters was analyzed (Figure 2B). Roles of the top‐ranking markers were clarified to infer the putative biological roles of each cluster, as listed in Table 2.

**TABLE 2:** Characteristics of peripheral immune subsets

**Clusters**	**Definition**	**Marker expression**
C01	Eosinophil	CD3^−^CD19^−^CD123^−^CD16^low^CD66b^+^CD11b^+^
C02	Eosinophil	CD3^−^CD19^−^CD123^−^CD16^low^CD66b^+^CD11b^+^
C03	Neutrophil	CD3^−^CD19^−^CD123^−^CD16^+^CD66b^+^CD11b^+^
C04	Neutrophil	CD3^−^CD19^−^CD123^−^CD16^+^CD66b^+^CD11b^+^
C05	Neutrophil	CD3^−^CD19^−^CD123^−^CD16^+^CD66b^+^CD11b^+^
C06	Neutrophil	CD3^−^CD19^−^CD123^−^CD16^+^CD66b^+^CD11b^+^
C07	Neutrophil	CD3^−^CD19^−^CD123^−^CD16^+^CD66b^+^CD11b^+^
C08	Neutrophil	CD3^−^CD19^−^CD123^−^CD16^+^CD66b^+^CD11b^+^
C09	Neutrophil	CD3^−^CD19^−^CD123^−^CD16^+^CD66b^+^CD11b^+^
C10	Neutrophil	CD3^−^CD19^−^CD123^−^CD16^+^CD66b^+^CD11b^+^
C11	Neutrophil	CD3^−^CD19^−^CD123^−^CD16^+^CD66b^+^CD11b^+^
C12	Neutrophil	CD3^−^CD19^−^CD123^−^CD16^+^CD66b^+^CD11b^+^
C13	Neutrophil	CD3^−^CD19^−^CD123^−^CD16^+^CD66b^+^CD11b^+^
C14	Neutrophil	CD3^−^CD19^−^CD123^−^CD16^+^CD66b^+^CD11b^+^
C15	Neutrophil	CD3^−^CD19^−^CD123^−^CD16^+^CD66b^+^CD11b^+^
C16	Neutrophil	CD3^−^CD19^−^CD123^−^CD16^+^CD66b^+^CD11b^+^
C17	Neutrophil	CD3^−^CD19^−^CD123^−^CD16^+^CD66b^+^CD11b^+^
C18	Neutrophil	CD3^−^CD19^−^CD123^−^CD16^+^CD66b^+^CD11b^+^
C19	Neutrophil	CD3^−^CD19^−^CD123^−^CD16^+^CD66b^+^CD11b^+^
C20	Neutrophil	CD3^−^CD19^−^CD123^−^CD16^+^CD66b^+^CD11b^+^
C21	Neutrophil	CD3^−^CD19^−^CD123^−^CD16^+^CD66b^+^CD11b^+^
C22	DC (cDC)	CD38^+^CD39^+^CD59^+^CD1c^+^CD11c^+^Tim3^+^CD45RO^+^HLA‐DR^+^
C23	DC (cDC)	CD38^+^CD39^+^CD45RA^+^CD11c^+^Tim3^+^CD45RO^+^CD16^+^HLA‐DR^+^
C24	Monocytes	CD14^+^CD38^+^CD39^+^CD11c^+^Tim3^+^CD45RO^+^HLA‐DR^+^CD11b^+^
C25	Monocytes	CD14^+^CD38^+^CD39^+^CD11c^+^CTLA‐4^+^Tim3^+^CD45RO^+^HLA‐DR^+^CD11b^+^
C26	Monocytes	CD14^+^CD38^+^CD39^+^CD11c^+^CD45RO^+^HLA‐DR^+^CD11b^+^
C27	DC (pDC)	CD123^+^CD38^+^CD59^+^CD11c^+^CD45RO^+^CD11b^+^
C28	NK cell	CD56^+^CD38^+^CD45RA^+^CD11c^+^Tbet^+^GranzymeB^+^CD11b^+^
C29	NK cell	CD56^+^CD38^+^CD45RA^+^CD11c^+^Tbet^+^GranzymeB^+^CD11b^+^
C30	B cell	CD19^+^CD38^+^CD39^+^CD45RA^+^CD1c^+^CCR7^+^HLA‐DR^+^
C31	B cell	CD19^+^CD38^+^CD39^+^CD45RA^+^CCR7^+^HLA‐DR^+^
C32	γδT	CD3^+^TCRd^+^CD45RO^+^Tbet^+^GranzymeB^+^
C33	CD8^+^ TEM	CD3^+^CD8^+^GranzymeB^+^
C34	CD8^+^ TEMRA	CD3^+^CD8^+^CD45RA^+^CCR7^−^Tbet^+^GranzymeB^+^
C35	CD4^+^ TEM	CD3^+^CD4^+^CD45RA^−^CCR7^−^CD45RO^+^Tbet^+^PD‐1^+^
C36	CD4^+^ TEM (Treg cell)	CD3^+^CD4^+^CD45RA^−^CCR7^−^CD45RO^+^CD27^+^CD25^+^CD28^+^
C37	CD4^+^ TCM	CD3^+^CD4^+^CD45RA^−^CCR7^+^CD45RO^+^CD27^+^CD28+CD127^+^
C38	CD4+ TEM	CD3^+^CD4^+^CD45RA^−^CCR7^−^CD45RO^+^CD27^+^CD127^+^
C39	CD4+ naïve T	CD3^+^CD4^+^CD45RA^+^CCR7^+^CD27^+^CD28^+^CD127^+^
C40	CD8+ naïve T	CD3^+^CD8^+^CD45RA^+^CCR7^+^CD27^+^CD28^+^CD127^+^

Using the expressions of PD‐1, chemokine C–C motif receptor 7 (CCR7), CD45RA, CD38 and HLA‐DR, we analyzed proportion of exhausted and activated CD4^+^ and CD8^+^ T cells in healthy volunteers and patients with lung diseases, respectively (Figure 2C–F). The proportion of activated CD4^+^ T cells was decreased in patients with LC, COPD and AECOPD, while increased in pneumonia (Figure 2C,E). The proportion of activated CD8^+^ T cells increased in patients with LC, indicating that the function of activated CD8^+^ T cells needs to be more thoroughly studied. It is noteworthy that significant exhaustion was significant in acute pneumonia (Figure 2D). Patients with acute pneumonia, stable pneumonia, acute asthma or stable asthma contained a small frequency of CD4^+^ Tex cells, with the large majority being activated CD4^+^ T cells. The frequencies of CD8^+^ T cells and activated CD8^+^ T cells significantly increased to verify the key roles of CD8^+^ T cells in LC (Figure 2D). The frequencies of CCR7^+^CD45RA^−^ CD4^+^ TCM and CCR7^−^CD45RA^−^ effector memory CD4^+^ T cells (CD4^+^ TEM) varied significantly among different groups (Figure 2D). The frequencies of effector memory CD8^+^ T cells (CD8^+^ TEM), effector CD8^+^ T cells, naïve CD8^+^ T cells or cytotoxic lymphocytes are shown in Figure 2E. The frequency of CD8^+^ TEM significantly increased in patients with COPD and AECOPD, and were very rare in LC group (Figure 2E). The distribution of transcription factors (CD137, T‐cell immunoglobulin and mucin domain‐containing protein 3 (Tim3), Oxford 40 ligand (OX40), eomesodermin (Eomes) and RORγ) and immunomodulatory molecules (CD40L, PD‐1, PD‐L1, TIGIT and CD59) are shown in Figure 2H.

### 3.2 T‐cell immune patterns among lung diseases

**FIGURE 3:**
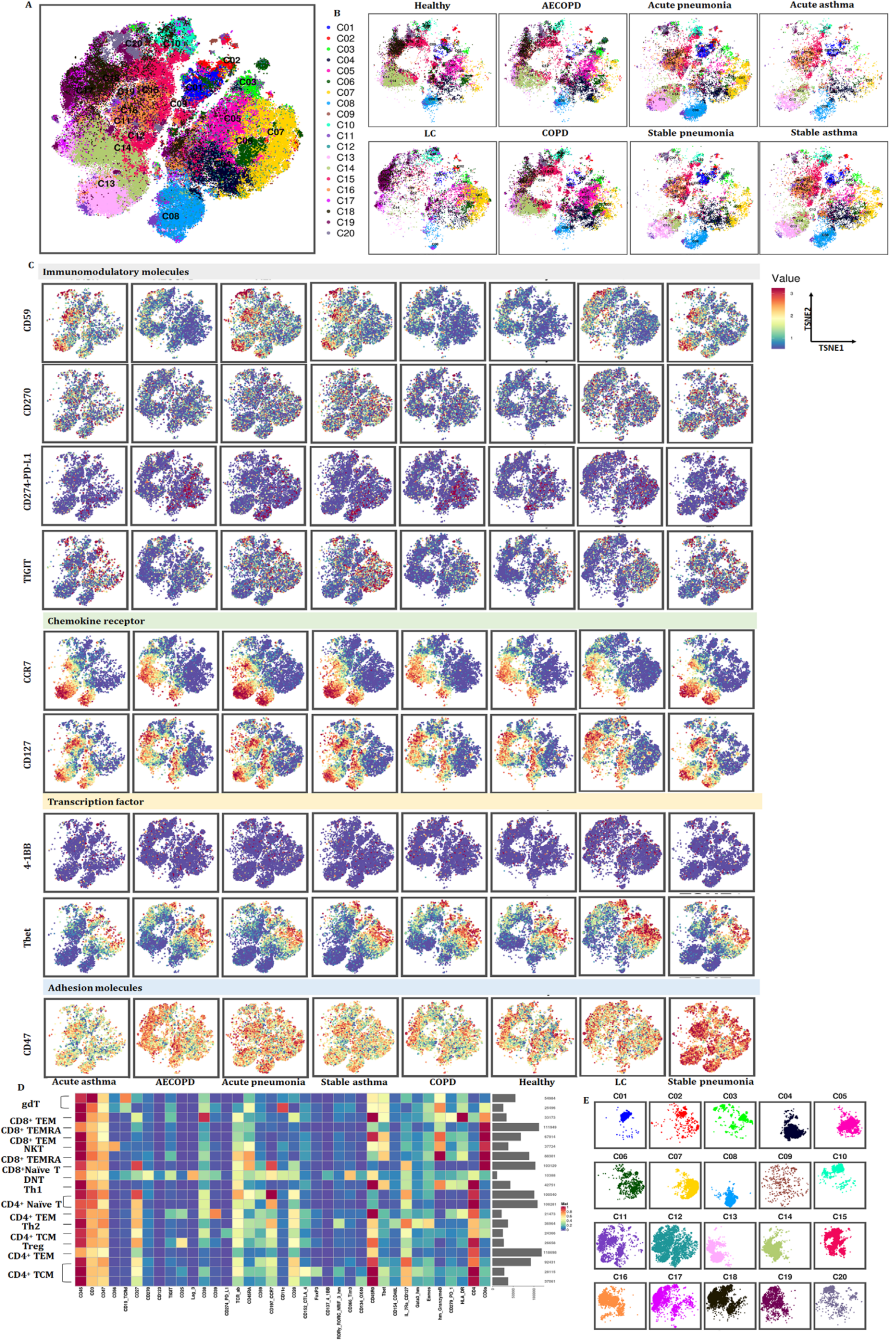
Identification of the peripheral T‐cell subsets. (A and B) Heatmap of T subsets in healthy controls and patients with lung diseases. We analyzed 20 clusters within T cells and showed the difference in the distribution of healthy controls and patients with lung diseases. (C) Distribution of immune molecules in healthy volunteers and patients with lung diseases. The distribution of transcription factors, chemokine receptors, adhesion molecules and immunomodulatory molecules in healthy volunteers and patients with acute pneumonia, stable pneumonia, stable asthma, acute asthma, COPD, AECOPD or LC are displayed. Distribution of other markers is shown in Figure S1. (D) Heatmap showing the expression levels of markers in T subsets. Clustering analysis for markers distinguished two CD4^+^ TEM subsets, three CD4^+^ TEM subsets, two CD8^+^ TEM subsets, two CD8^+^ TEMRA cell subsets, pDCs, two cDC subsets, and others. (D) TSNEs showing the distributions of markers in T subsets. The distributions of analyzed clusters in (D) are displayed

Our previous studies defined metabolic insufficiency of exhausted CD4^+^ or CD8^+^ T cells in patients with AECOPD. In the current study, our further analysis reveals the diversity of T cells, with approximately 20 coarse‐grained cell types in total (Figure 3A). Cluster 13, which is defined as CD4^+^ naïve T, was significant in patients with acute pneumonia, stable pneumonia, acute asthma and asthma as compared with healthy volunteers, patients with AECOPD, COPD and LC. Observed extensive overlaps in cell‐type identities are listed in Table 3. The distribution of PBIC subsets of healthy controls and patients with AECOPD, COPD, acute pneumonia, stable pneumonia, acute asthma and stable asthma is shown in Figure 3B. The distribution of immunomodulatory molecules, chemokine receptors, transcription factors and adhesion molecules is shown in Figure 3C and Figure S1. We firstly measured TEM, TCM, Th1, Th2 cells and T_reg_, and identified eight TEM subsets, including two CD8^+^ T cells, two CD8^+^ terminal differentiation effector T cells (TEMRA), two CD4^+^ TEM and one Th1 and Th2 subsets. There were also three CD4^+^ TCM subsets, two γδT subsets, CD8^+^ naïve T, two CD4^+^ naïve T and Treg subsets (Figure 3D and Table 3). The distribution of these subsets is shown in Figure 3E.

**TABLE 3:** Characteristics of peripheral CD3^+^ T subsets

**Cluster**	**Definition**	**Marker expression**
C01	γδT	CD3^+^TCRd^+^ CD45RA^−^CCR7^−^CD45RO^+^Tbet^+^GranzymeB^+^
C02	γδT	CD3^+^ TCRd^+^CD45RA^+^CCR7^−^CD11c^+^CD45RO^+^Tbet^+^GranzymeB^+^
C03	CD8^+^ TEM	CD3^+^CD8a^+^TCRab^+^CD45RA^−^CCR7^−^CD45RO^+^CD27^+^CD38^+^CD28^+^ Tbet^+^Emos^+^GranzymeB^+^PD‐1^+^HLA‐DR^+^
C04	CD8^+^ TEMRA	CD3^+^CD8a^+^TCRab^+^CD45RA^+^CCR7^−^CD45RO^−^GranzymeB^+^
C05	CD8^+^ TEM	CD3^+^CD8a^+^TCRab^+^CD45RA^−^CCR7^−^CD45RO^+^Tbet^+^GranzymeB^+^
C06	NKT	CD3^+^CD56^+^CD8a^+^TCRab^+^CD45RA^+^CCR7^−^CD45RO^+^Tbet^+^GranzymeB^+^
C07	CD8^+^ TEMRA	CD3^+^CD8a^+^TCRab^+^CD45RA^+^CCR7^−^CD45RO^−^Tbet^+^GranzymeB^+^HLA‐DR^+^
C08	CD8^+^ naïve T	CD3^+^CD8a^+^TCRab^+^CD45RA^+^CCR7^+^CD45RO^−^CD27^+^CD127^+^
C09	DNT	CD3^+^CD270^+^TIGIT^+^ LAG3^+^TCRab^+^CD45RA^−^CCR7^+^CD45RO^+^Tim3^+^
C10	CD4^+^ TEMRA (Th1)	CD3^+^CD4^+^TCRab^+^CD45RA^−^CCR7^−^CD45RO^−^Tbet^+^GranzymeB^+^HLA‐DR^+^
C11	CD4^+^ TCM (Th2)	CD3^+^CD4^+^TCRab^+^CD45RA^−^CCR7^+^CD45RO^+^CD27^+^CD127^+^GATA3^+^Eomes^+^
C12	CD4^+^ TCM	CD3^+^CD4^+^TCRab^+^CD45RA^−^CCR7^+^CD45RO^+^CD27^+^CD127^+^
C13	CD4^+^ naïve T	CD3^+^CD4^+^TCRab^+^CD45RA^+^CCR7^+^CD45RO^−^CD27^+^CD28^+^CD127^+^
C14	CD4^+^ naïve T	CD3^+^CD4^+^TCRab^+^CD45RA^+^CCR7^+^CD45RO^−^CD27^+^CD38^+^CD127^+^
C15	CD4^+^ TEM	CD3^+^CD4^+^TCRab^+^CD45RA^−^CCR7^−^CD45RO^+^CD27^+^CD127^+^
C16	CD4^+^ TCM	CD3^+^CD4^+^TCRab^+^CD45RA^−^CCR7^+^CD45RO^+^CD27^+^CD28^+^CD127^+^
C17	CD4^+^ TCM	CD3^+^CD4^+^TCRab^+^CD45RA^−^CCR7^+^CD45RO^+^CD27^+^CD28^+^CD127^+^CTLA4^+^
C18	CD4^+^ TCM	CD3^+^CD4^+^TCRab^+^CD45RA^−^CCR7^+^CD45RO^+^CD27^+^CD28^+^CD127^+^OX40^low^Tbet^low^
C19	Treg	CD3^+^CD4^+^TCRab^+^CD45RA^−^CCR7^+^CD45RO^+^CD27^+^CD25^+^
C20	CD4^+^ TEM	CD3^+^CD4^+^TCRab^+^CD45RA^−^CCR7^−^CD45RO^+^

### 3.3 Frequency of PBIC subset clusters among lung diseases

**FIGURE 4:**
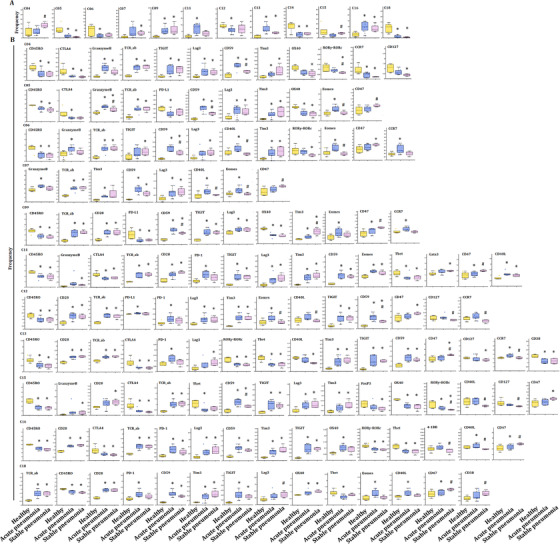
T‐cell immune patterns across healthy volunteers and patients with acute pneumonia and stable pneumonia showed the potential roles of exhausted T subsets and effector CD4^+^ TCM in acute pneumonia. (A) Boxplots showing the frequencies of the indicated T‐cell clusters that have alteration in the PBMC of healthy controls, acute pneumonia and stable pneumonia patients. Twelve CD3^+^ T subsets were defined to be significantly altered. The markers expression on theses subsets is shown in Table 3. The boxplots of other T clusters are shown in Figure S2A. (B) Boxplots showing the frequencies of significantly altered markers in the significantly altered subsets of (A). The expression levels of exhaustion markers, including TIGIT, PD‐L1, LAG3, Tim3, OX40, were analyzed in significantly altered T subsets in acute pneumonia and stable pneumonia

The frequencies of several clusters of T subsets significantly altered between patients with acute pneumonia or stable pneumonia, rather than specific clusters of T subsets. The frequency of clusters 09 and16 significantly increased in acute pneumonia and stable pneumonia, as compared with healthy individuals (Figure 4A). The frequency of cluster 07 significantly increased in acute pneumonia, as compared with healthy group. The frequency of clusters 05, 14 and 18 significantly decreased in acute pneumonia, as compared with healthy group. The frequency of clusters 04 and 15 in T cells of patients with stable pneumonia was significantly higher than those with acute pneumonia (Figure 4A). The frequencies of the other clusters are shown in Figure S2A.

The frequency of cluster 9 was identified as DNT cell with markers of CD3^+^ TCRd^+^CD45RA^+^CCR7^−^CD11c^+^CD45RO^+^ T‐box expression in T‐cell (Tbet)^+^ Granzyme B^+^ (Table 3), and was one of the increased subsets of CD3^+^ T cells in acute pneumonia. The expression levels of CD59, CD28, CCR7, CD270, Tim3, Eomes, lymphocyte activation gene‐3 (LAG3), TCRab and TIGIT were higher, while that of OX40, CD45RO, PD‐L1 and CD25 lower in cluster 09 in patients with acute pneumonia. CCR7 in cluster 09 increased significantly only in peripheral CD3^+^ T cells of the patients with acute pneumonia (Figure 4B).

### 3.4 Tex subsets in acute pneumonia and new mechanisms of immune modulation

Of subsets of CD3^+^ T cells, the frequencies of cluster 06 identified as NKT cell with markers of CD3^+^ CD3^+^CD56^+^CD8a^+^TCRab^+^CD45RA^+^CCR7^−^CD45RO^+^Tbet^+^GranzymeB^+^ (Table 3) and cluster 12 as CD4^+^ TCM with markers of CD3^+^CD4^+^TCRab^+^CD45RA^−^CCR7^+^CD45RO^+^CD27^+^CD127^+^ significantly decreased in acute pneumonia. The expression levels of CD40L, Eomes and Granzyme B were significantly higher in patients with acute pneumonia, which indicates that the differentiation promotion of CD4^+^ T cells into Th17 and IgE secretion might be inhibited.^20^

**FIGURE 5:**
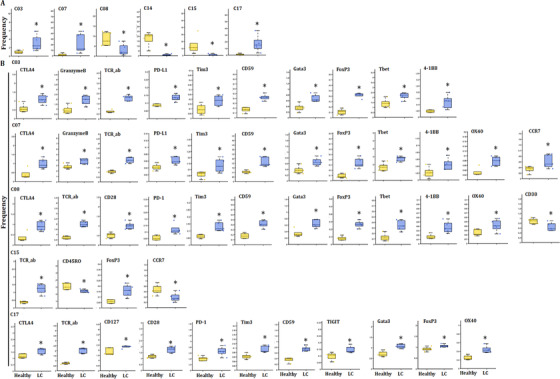
T‐cell immune patterns across healthy volunteers and patients with lung cancer (LC). (A) Boxplots showing the frequencies of the indicated T‐cell clusters that have alteration in the PBMC of healthy controls and patients with LC. Six T subsets were defined to be significantly altered. The markers expression on theses subsets is shown in Table 3. The boxplots of other T clusters are shown in Figure S2B. (B) Boxplots showing the frequencies of significantly altered markers in the significantly altered subsets of (A). The expression levels of exhaustion markers, including TIGIT, PD‐L1, LAG3, Tim3, OX40, were analyzed in significantly altered T subsets in patients with LC, compared with healthy volunteers

Clusters 09 and 16, which identified to be DNT and CD4^+^ TCM, respectively, significantly increased in patients with acute pneumonia and stable pneumonia with an enhanced expression of Lag3 and Tim3, which were identified to be the exhausted clusters. The expression level of CD45RO decreased in these two clusters. The frequency of cluster 18 as another decreased subset of CD3^+^ T cells reduced in acute pneumonia. Cluster 18 was identified as CD4^+^ TCM with markers of CD3^+^CD4^+^TCRab^+^CD45RA^−^CCR7^−^CD45RO^+^ (Table 3 and Figure 5). The expression levels of CD27, CD28, CD59, OX40, CD40L, CD270, PD‐1, Tim3, EOME, HLA‐DR, TCRab and TIGIT were higher, while CD45, CD45RO and Tbet were lower in cluster 18 of patients with acute pneumonia. CD40L and Tbet mediated activation of T cells and contributed to effector T‐cell responses.^21^, ^22^ The expression of Eomes increased in tumour‐infiltrating CD8^+^ T cells, especially in PD‐1^+^ Tim‐3^+^‐depleted CD8^+^ T cells, indicating the contribution in T‐cell exhausting.^18^, ^23^ The increase of PD‐1 and Eomes and the decrease of Tbet in cluster 18 of patients with LC indicated the exhaustion of CD3^+^ T subsets during acute pneumonia patients, which enriched the mechanisms of immune modulation.

### 3.5 Pathogenic effector CD4^+^ TCM and lung cancer

Alteration of frequencies of T subsets was further analyzed in the T‐cell subsets in LC, as compared with the healthy group. The frequency of clusters 03, 07 and 17 significantly increased in LC (Figure 5A), of which cluster 07 was significantly higher than that in COPD or AECOPD (Figure S3), and cluster 17 was higher in acute pneumonia, stable pneumonia, acute asthma or stable asthma (Figure S3). The frequencies of clusters 08 and 15 significantly decreased in LC, as compared with healthy group (Figure 5A), of which cluster 8 in acute pneumonia and stable pneumonia was higher than LC (Figure S3). The frequency of cluster 15 in LC is lower than that in AECOPD, COPD, stable pneumonia, acute asthma or stable asthma. The frequency of cluster 04 in stable pneumonia, acute asthma or stable asthma was higher than that in LC. The frequency of cluster 05 in LC was significantly higher than that in acute pneumonia and stable pneumonia. The frequency of cluster 06 in LC was higher than that in acute pneumonia, stable pneumonia or stable asthma, which could be relevant with immune suppression. The frequencies of clusters 11 or 13 in LC were significantly lower than that in acute pneumonia or in stable pneumonia. The frequency of cluster 16 was higher in acute pneumonia, stable pneumonia, acute asthma or stable asthma, while that of cluster 18 was lower in pneumonia, stable pneumonia or stable asthma, as compared with that in LC (Figure S3).

The cluster 15 was identified as CD4^+^ TEM with markers of CD3^+^CD4^+^TCRab^+^CD45RA^−^CCR7^−^CD45RO^+^CD27^+^CD127^+^ (Table 3) and decreased as LC‐specific subset in CD3^+^ T cells. The expression levels of CD45RA, CD270, forkhead box P3 (Foxp3) and TCRab increased, whereas CD45RO and CCR7 decreased in cluster 15 in LC (Figure 5A). The cluster 17 was identified as CD4^+^ TCM with markers of CD3^+^CD4^+^TCRab^+^CD45RA^−^CCR7^+^CD45RO^+^CD27^+^CD28^+^CD127^+^CTLA4^+^(Table 3) and increased as LC‐specific CD3^+^ T‐cell subset (Figure 5A). The expression levels of CD27, CD28, CD45RA, CD56, CD59, OX40, CTLA4, CD270, PD‐1, Tim3, Foxp3, GATA binding protein 3 (GATA3), CD127, TCRab and TIGIT increased in cluster 17 of CD3^+^ T cells in LC (Figure 5A). CD45RA and GATA3 levels increased in cluster 17 in LC, indicating the specificity of LC (Figure 5B). The frequencies of other clusters are shown in Figure S2B.

### 3.6 PBIC features in AECOPD and COPD

**FIGURE 6:**
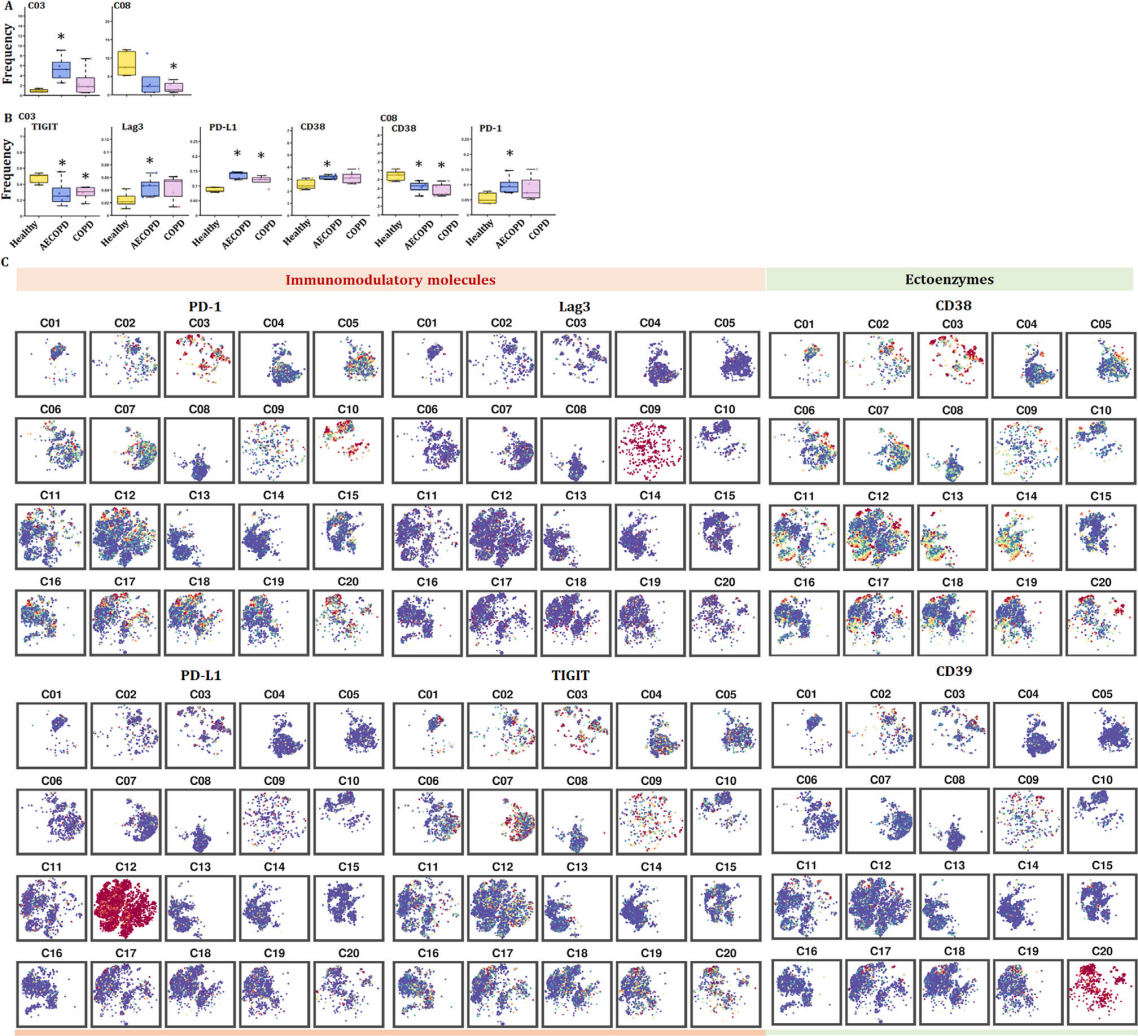
T‐cell immune patterns across healthy volunteers and patients with AECOPD and COPD. (A) Boxplots showing the frequencies of the indicated T‐cell clusters that have alteration in the PBMC of healthy controls, AECOPD and COPD patients. Two T subsets were defined to be significantly altered. The markers expression on theses subsets is shown in Table 3. The boxplots of other T clusters are shown in Figure S2C. (B) Boxplots showing the frequencies of significantly altered markers in the significantly altered subsets of (A). The expression levels of significantly changing markers in these subsets, such as CD38, were analyzed in significantly altered T subsets in patients with AECOPD and COPD, compared with healthy volunteers. (C) TSNEs showing the distributions of immunomodulatory molecules in every T subset. The distribution of significantly changing markers is shown in every CD3^+^ T‐cell subset in Figure 3A

The frequencies of several clusters of T subsets were changed in T‐cell subsets in AECOPD and COPD, as compared with healthy volunteers. The frequency of cluster 08 significantly decreased in COPD, while cluster 03 was significantly higher than in LC or healthy group, respectively (Figure 6A). The frequency of clusters 05 and 06 in COPD was significantly higher than in acute pneumonia (Figure S3), clusters 07 and 08 in stable pneumonia and cluster 09 in acute pneumonia than in AECOPD, and cluster 12 in AECOPD higher than acute pneumonia, respectively. The frequencies of cluster 13 in acute pneumonia and cluster 16 in acute pneumonia, stable pneumonia, stable asthma and acute asthma were higher than in AECOPD and COPD, respectively. The frequency of cluster 18 in AECOPD and COPD was higher compared with acute pneumonia, stable pneumonia or stable asthma (Table 3 and Figure S3).

The frequency of cluster 3 increased in AECOPD, acute asthma and LC. The expression of CD38 in cluster 03 of CD3^+^ T cells significantly increased in AECOPD, as compared with that in healthy group (Figure 6B). CD38 was the main mechanism of acquired resistance to PD‐1/PD‐L1 blocking, leading to the reservation of CD8^+^ T‐cell antivirus activation. The frequencies of other clusters are shown in Figure S2C.

### 3.7 Comparison between asthma and pneumonia

**FIGURE 7:**
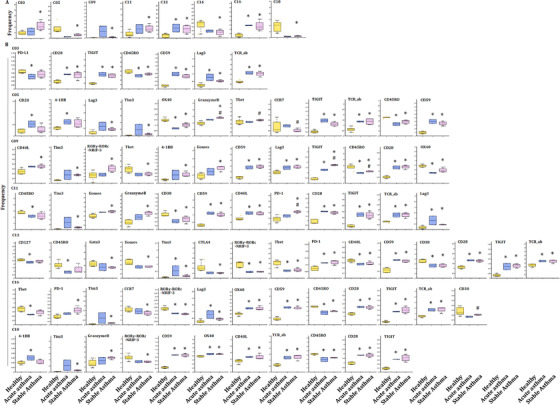
T‐cell immune patterns across healthy volunteers and patients with acute asthma and stable asthma. (A) Boxplots showing the frequencies of the indicated T‐cell clusters that have alteration in the PBMC of healthy controls, acute asthma and stable asthma patients. Eight T subsets were defined to be significantly altered in acute asthma or stable asthma patients, compared with healthy volunteers. The markers expression on theses subsets is shown in Table 3. The boxplots of other T clusters are shown in Figure S2C. (B) Boxplots showing the frequencies of significantly altered markers in clusters of (A). Expression levels of significantly changing markers, including PD‐L1, TIGIT and LAG3, were analyzed in significantly altered T subsets in patients with acute asthma or stable asthma, compared with healthy volunteers

Alterations of T‐subset frequencies were further analyzed between asthma and healthy, between acute and chronic states and between asthma and pneumonia. The frequency of clusters 13 and 16 significantly increased in acute asthma and stable asthma (Figure 7A), as well as in acute pneumonia and stable pneumonia (Figure 4A), as compared with that in healthy volunteers. The expression levels of CD27, CD28, CD59 and TCRab were higher, while CD38, CD39, CD45, CD45RO, RORγ and Tbet were lower in cluster 13 in acute pneumonia, stable pneumonia, acute asthma and stable asthma, while the levels of PD‐1 in pneumonia and acute asthma were higher, suggesting T cells of those patients may be more exhaustive. The expression levels of CD28, CD59, LAG3, TCRab and TIGIT were higher, while CD45, CD45RO, RORγ, OX40 and Tbet lower in cluster 16 in acute pneumonia, stable pneumonia, acute asthma and stable asthma (Figures 4B and 7B). The frequencies of the other clusters are shown in Figure S2D.

### 3.8 PBIC bulk RNA‐seq and DEGs

**FIGURE 8:**
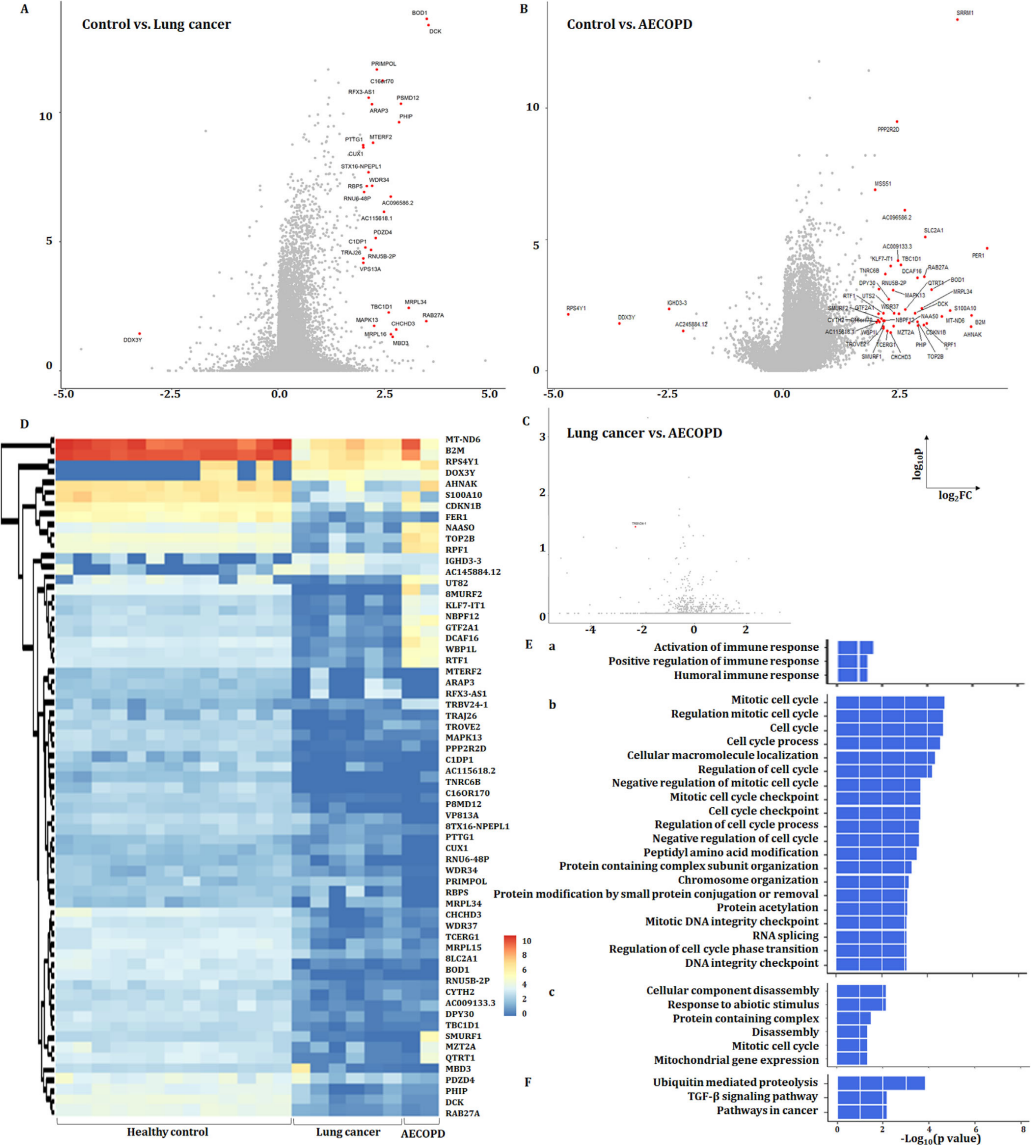
Gene expression alterations in healthy controls, patients with lung cancer (LC) or AECOPD. (A) Differential genes in healthy controls and patients with LC. (B) Differential genes in healthy controls and patients with AECOPD. (C) Differential genes in patients with LC and patients with AECOPD. (D) Heatmap showing the differential genes from (A)–(C) in individuals. (E) GO analysis for biological processes upregulated in healthy controls compared with patients with LC (a); downregulated in patients with LC compared with healthy controls (b); downregulated in patients with AECOPD compared with healthy controls (c). (F) GO analysis for KEGG downregulated in patients with LC compared with healthy controls

We further compared the gene expressions among the control group, patients with LC or with AECOPD as a representative of no‐cancer, as the repeatability of acute exacerbation was considered as a critical factor to initiate and accelerate the COPD–LC transition.^3^, ^24^, ^25^, ^26^ One gene was upregulated and 30 genes downregulated in LC (Figure 8A), and four genes downregulated and 39 genes upregulated in AECOPD (Figure 8B), as compared with healthy group, the expression of *TRBV24‐1* was significantly downregulated in LC, compared with AECOPD (Figure 8C). The expression of DEGs in PFICs of the healthy group is shown in Figure 8D. Three upregulated signal pathways mainly about immune response regulation (Figure 8Ea) and 20 downregulated signal pathways including cell cycle and DNA integrity regulation (Figure 8Eb) in LC as well as six downregulated signal pathways in AECOPD (Figure 8Ec) were found, as compared with healthy group. KEGG analysis showed that three signalling pathways including ubiquitin‐mediated proteolysis, TGF‐β signalling pathways and pathways in cancer were downregulated in LC, as compared with the healthy sample (Figure 8F).

### 3.9 PBIC scRNA‐seq and heterogeneity

**FIGURE 9:**
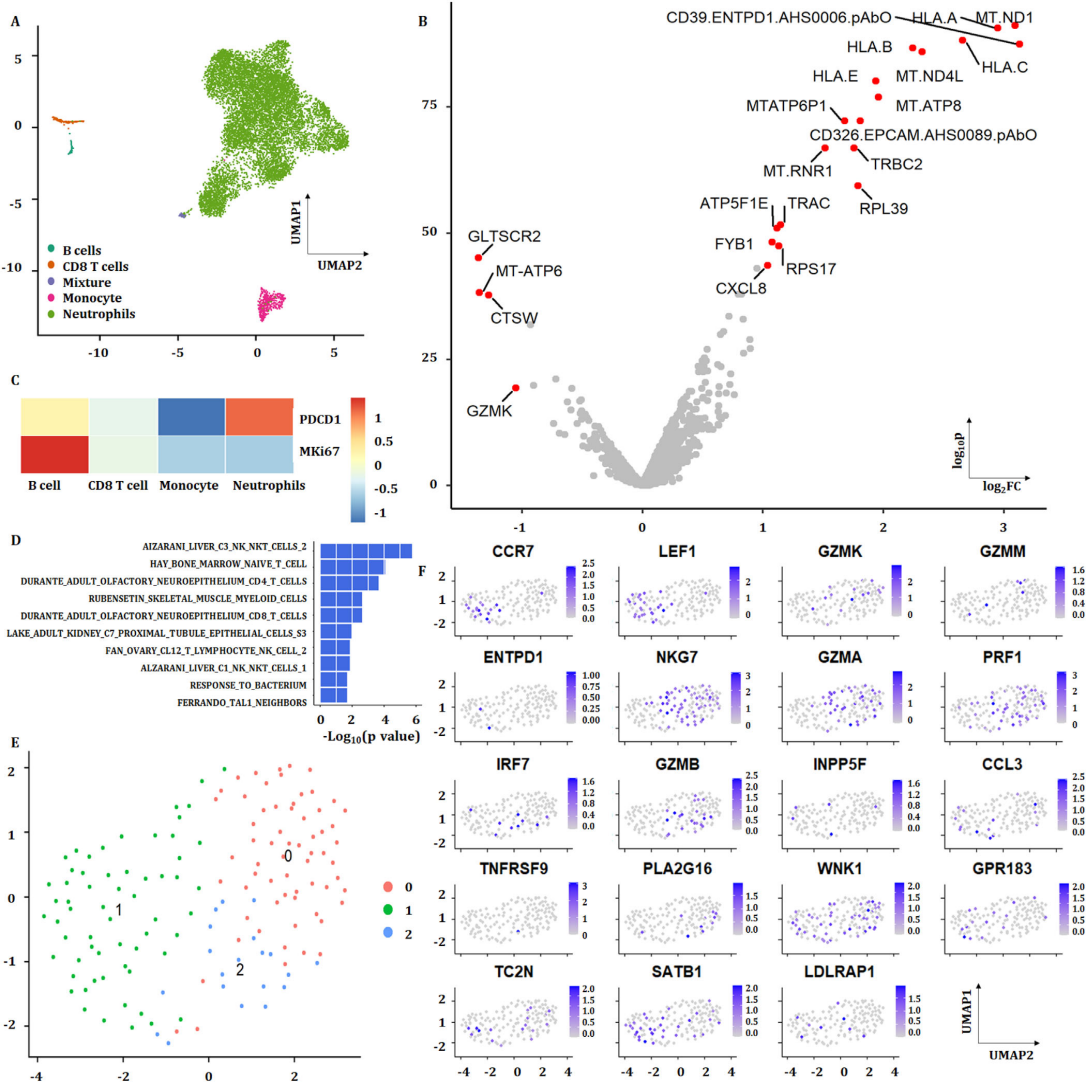
Single‐cell sequencing analysis showing the cell type distribution and gene expression in acute pneumonia patients. (A) Cluster distribution in the peripheral blood cells in patients with acute pneumonia. (B) Differential genes in acute pneumonia patients and healthy controls; single‐cell sequencing data from the published article. (C) PDCD1 and MKi67 gene expression in cell types of the peripheral blood from acute pneumonia patients. (D) GO analysis for biological processes upregulated in CD8^+^ T cells from acute pneumonia patients compared with healthy controls. (E) Three clusters were analyzed from CD8^+^ T cell. (F) Distribution of genes, such as CCR7, GPR183 and others, in acute pneumonia patients are shown

The scRNA‐seq of PBICs was performed in various groups, about 10 000–20 000 single cells per sample and three to four samples per group, as shown in Figure 9. The results showed that neutrophils and subtypes were the main components of PBICs in acute pneumonia (Figure 9A). A subset of genes was identified with high cell‐to‐cell variation. The downstream analyses represented the biological signals in downstream analyses. Of 10 323 cells, about 400–500 detected genes per cell were performed for downstream analyses. The expression of four genes decreased and 18 genes increased in acute pneumonia patients (Figure 9B). The expression of *PDCD1*(*PD‐1*) and *MKI67* in acute pneumonia was analyzed (Figure 9C). One hundred forty‐five CD8^+^ T cells identified from three patients were compared with 308 CD8^+^ T cells from the health donor (Ref: 0 https://support.10xgenomics.com/single‐cell‐gene‐expression/datasets/1.1.0/pbmc3k?) With rank‐sum test, the genes with FDR <0.05 and log2 fold change >1 are shown in volcano plot and their corresponding enrichment analyses were analyzed with GSEA (Figure 9D). Three clusters were analyzed from CD8^+^ T cell (Figure 9E). The expression of G protein‐coupled receptor (GPR) 183 and CCR7 was positive on CD8^+^ T cells in acute pneumonia.

### 3.10 Potential mechanisms of TC–PBIC interactions

**FIGURE 10:**
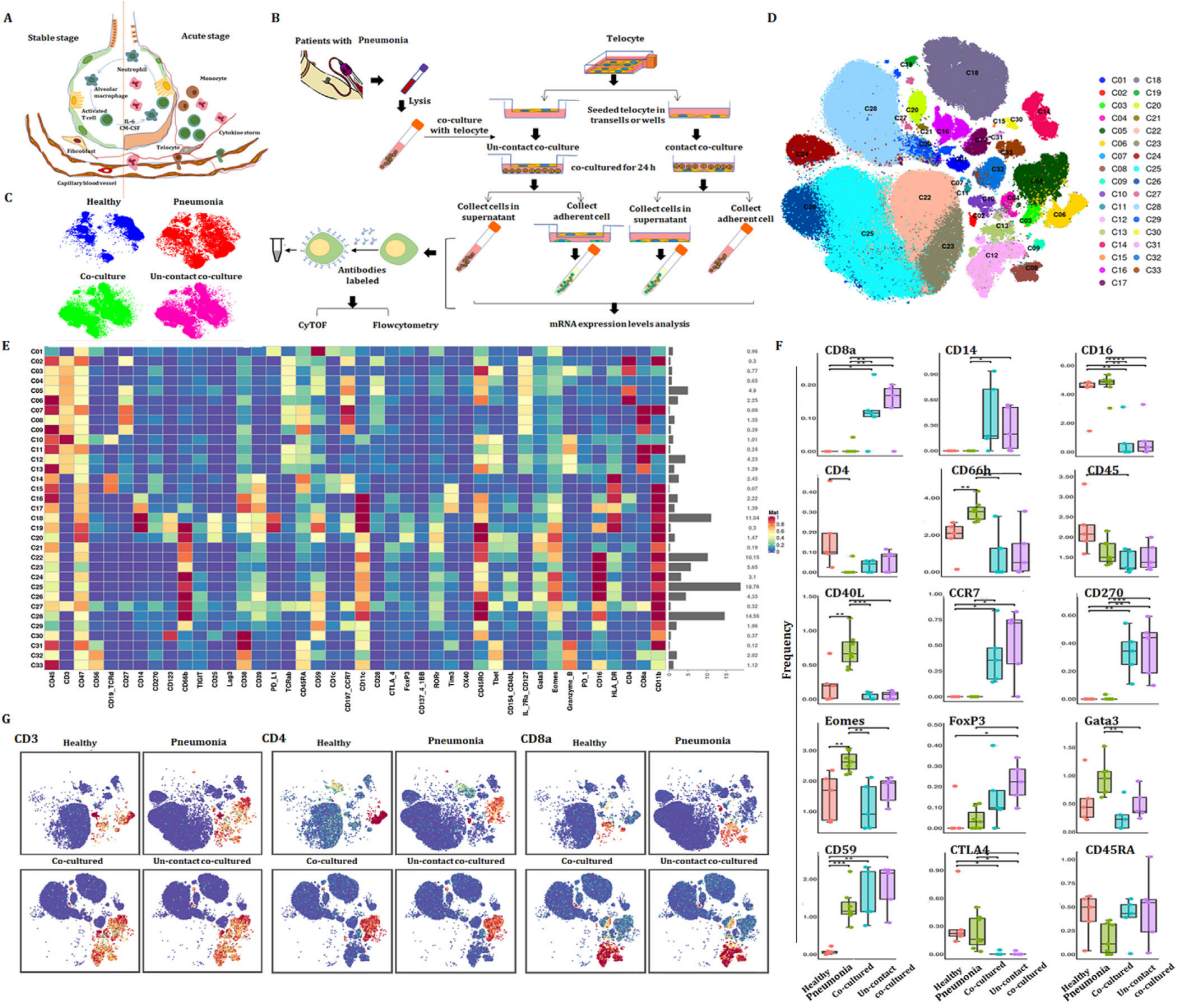
Effect of interstitial cells on immune cells in acute pneumonia patients. (A) Diagram of the interaction between immune cells and other types of cells in tissue in acute stage and stable stage of pneumonia. (B) Experimental design of the co‐culture of peripheral leukocytes of the patients with acute pneumonia and TCs. (C) The distribution of CD45^+^ cells in healthy volunteers, patients with acute pneumonia, and leukocytes co‐cultured with TCs in contact or noncontact form. (D) The distribution of CD45^+^ subsets. (E) The expression of immune markers. (F) The frequency of immune markers in the patients with acute pneumonia and decreased after being co‐cultured with TCs. (G) The distribution of CD3, CD4 and CD8a in different groups

In the acute stage of pneumonia, the interaction between immune cells and other types of cells in tissue plays a key role in the development of the disease (Figure 10A), as the cell–cell communication was suggested to be one of the critical approaches to translate the signals into the tissue and to maintain microenvironmental haemostasis.^27^, ^28^ TCs are new type of interstitial cells and their intercellular communication plays important roles in acute lung injury.^28^, ^29^ The protocol of co‐culture between PBICs from patients with acute pneumonia and primary TCs from lung sections is shown in Figure S5. Alteration of immune cell types and immune function were detected using CyTOF (Figure 10B). Figure 10C shows the distribution of CD45^+^ cells in healthy, acute pneumonia, PBIC‐TC contact co‐culture and PFIC‐TC noncontact co‐culture. The distribution of CD45^+^ subsets and the expression of immune markers are shown in Figure 10D and Figure 10E, respectively. CD40L and Eomes significantly increased in acute pneumonia and decreased after being co‐cultured with TCs (Figure 10F). The expression levels of CD8a, CD14, CD270 and CD 59 were increased, while CD16 and CTLA4 were decreased compared with patients with pneumonia. The expression levels of CD66, CD40L and GATA3 were increased in patients with pneumonia, whereas reversed after being co‐cultured with TCs. The distribution of CD3, CD4 and CD8a in different groups is shown in Figure 10G.

## 4 DISCUSSION

Exhaustion occurs in CD8 T cells, CD4 T cells and other immune cells.^30^, ^31^ Although the mechanisms involved in the initial T‐cell exhaustion remain underappreciated, the persisting antigen stimulation plays a decisive role in the development of T‐cell exhaustion. Of those stimuli, the co‐stimulatory signalling may be improperly dampened or enhanced to contribute to T‐cell exhaustion. Our results showed that the frequencies of CD8^+^ T cells and activated CD8^+^ T cells increased in patients with LC. CD8^+^ T cells are the major antitumour effector cells.^32^ Chemotherapy increased the proliferation of effector CD8^+^ T cells, while had limited effect on patient prognosis.^33^ In patients with stable asthma, activated CD4^+^ T cells enriched, expressed and secreted proinflammatory cytokines, including IL‐17 and TNF α.^34^ CD8^+^ TEMs have the ability to persist for years and kill tumour and infected cells, to protect against LC and be the predictive factor of early response to postradiotherapy lung metastasis.^35^, ^36^, ^37^ The downregulation of naïve CD8^+^ T cells and CTL happens in chronic lung diseases including LC and COPD, of which naïve CD8^+^ T cells can induce systemic antitumour immunity and inhibit tumour growth.^38^

The number of CD4^+^ TCM and CD4^+^ TEM varied among lung diseases, of which quiescent CD4^+^ TEMs are present within the microenvironment of human LC and contribute to tumour cell apoptosis by releasing inflammatory mediators, secreting IFN‐γ and induce cell proliferation.^39^ Memory T cells induction or maintenance could be a novel therapy strategy.^40^, ^41^ We found the exhaustion of CD4^+^ TEM in patients with acute pneumonia accompanied with a decrease of CCR7^+^CD45RA^+^ naïve T cells (CD38^+^HLA‐DR^+^). The TEM‐increased immune states existed in patients with stable and acute asthma. The epigenetic landscape of Tex cells in cancer or chronic infections was described by assay for transposase‐accessible chromatin with high‐throughput sequencing (ATACseq), which is independent upon infective pathogens like virus or bacteria.^42^ Our current study shows that the parts of PBIC clusters or markers had the overlap among lung diseases.

CD4^+^ T‐cell recognition of neoantigens is frequent, including cytotoxic Th1 cells, Th17 cells with an early memory and/or a stem cell‐like phenotype and IL‐9‐producing CD4^+^ Th (Th9) cells. Eomes upregulation drives the effector development of Th9 cells.^43^, ^44^ High expression of Tbet drives the short‐lived T‐cell development and thus is closely associated with exhaustion. Exhausted CD4^+^ T cells biased expression of inhibitory molecules like B‐ and T‐lymphocyte attenuator, CTLA‐4, CD200 and PD‐1.^45^ Distinct combinations of inhibitory and co‐stimulatory pathways could lead to selective targeting of exhausted CD4^+^ and/or CD8^+^ T cells, while common inhibitor receptors include Lag3 and Tim3.^46^ In our current study, we identified DNT and CD4^+^ TCM expressed increased Lag3 and Tim3 in acute pneumonia and stable pneumonia. These results indicated the exhaustion in acute pneumonia. The CD8^+^ T‐cell exhaustion was inversely associated with the CD4^+^ T‐cell co‐stimulation signature and predicts a better prognosis in multiple autoimmune diseases.^47^CTLA‐4 expresses on activated T cells and is the receptor for B7 family. CTLA‐4 delivers negative co‐stimulatory signalling to T cells inhibiting TCR‐ and CD28‐mediated signal processes by interacting with B7.^6^ Memory T cells respond to respiratory pathogens and persist after pathogen clearance.^48^ Predominant memory T‐cell populations and effector memory phenotypes (TEM, CD45RA^−^CCR7^−^) of CD4^+^ and CD8^+^ T cells were contained in lung tissues and blood. Virus‐specific T cells in tissues express CD69, which maintains tissue haemostasis and shows lack of re‐circulation.^49^ The markers associated with tissue progress (CCR7) and immunoregulatory molecules (PD‐1, TIGIT, CTLA4) are essential for regulating inflammatory responses of memory T cells.^50^ The controlling of T‐cell exhaustion could affect the clinical outcome of autoimmunity.^51^ Persistent T‐cell receptor stimulations promote T‐cell exhaustion, which can be prevented in human CD8^+^ T cells by co‐stimulation via CD2. Nanoparticle‐based immune checkpoint inhibitor therapy, including PD‐1 or PD‐L1 blockers, increases the local concentration of immune checkpoint inhibitors while reduces the side effects in anti‐tumor therapies.^52^ The presence of CD4^+^ T‐cell co‐stimulation modules was correlated with interferon and ribavirin combination treatment for chronic hepatitis C virus infection.^53^

CTLA4, Tim3, Foxp3, GATA3, CD127, TCRab and TIGIT increased in cluster 17 of CD3^+^ T cell in patients with LC, of which CTLA4 expressed in tumour CD8 T cells associated with PD‐1‐mediated T‐cell dysfunction and efficacy of the checkpoint block immunotherapy in cancers.^54^, ^55^ Tim‐3 has the relationship with PD‐1 and its blocking enhances antitumour immunity and inhibits tumour growth.^56^ Foxp3 promotes tumour growth and metastasis by Wnt/β‐catenin activation and inducing epithelial mesenchymal transformation.^57^ The mutation of GATA3 drives tumour growth.^58^ TIGIT binds to CD155 or CD112 on the surface of tumour cells and antigen presenting cells in tumour microenvironment. A recent research reported that TIGIT could be a promising new target for tumour immunotherapy.^58^ These evidences indicate that cluster 17 should be a critical CD3^+^ T subset in patients with LC.

The T‐cell exhaustion was studied in chronic infections or cancer where antigen stimulation persists, while our results suggest new clusters of Tex subset in acute pneumonia. The heterogeneity within the exhausted lineage also exists with the development of exhaustion. CD8 Tex cells arise from the pool of KLRG‐1^lo^ CD8 Teff cells to induce CD127^+^ memory precursor cells.^59^ Those precursors develop into exhausted cells with persistent antigen stimulation and prolonged inflammation in chronic infections and cancers.^60^ Thus, the Tex cells could form from the precursors with memory potentials in the effector pool, which increases in acute inflammation. The activation of CD28 contributes to memory of T‐cell responses.^61^ CD59 as an important complement regulator inhibits the formation of membrane attack complex and mediates the signal transduction and activation of T lymphocytes.^62^ The altered cluster 09 is supposed to give the benefit for the patients with pneumonia, as PD‐L1 is a co‐suppression signal.^63^ The function of cluster 09 seems more complex to present the OX40 signalling pathway, to maintain CD4 memory response, CCR7 activation and lymphocyte homeostasis.^50^, ^64^, ^65^

Altered responsiveness for the same cluster or same marker from different lung diseases was found in the current study. For instance, the expression levels of CD45 and CD45RO in cluster 03 were lower in CD3^+^ T cells of the patients with LC and AECOPD, or asthma. The expression of CD47 in cluster 03 significantly increased in CD3^+^ T cells of patients with stable pneumonia. The elevated levels of 59 as essential complement regulatory protein in acute and stable pneumonia and asthma demonstrated that those diseases have strong capacities of systemic protection against complement attack. Such capacity can be developed during the progression of the disease, or in response to pathogens. CD59 can be an infection‐ or allergy‐associated biomarker or therapeutic target for drug development. Our data show that systemic immune cells like CD3^+^ T cells are activated to regulate various phases of the immune response through binding of increased CD154 (CD40‐ligand) to CD40 in acute pneumonia, as another indication in response to pathogens. Such CD154‐dependent responses were low in the patients with LC, AECOPD and COPD. Tim3 was identified as a receptor expressed on IFN‐γ‐producing CD4^+^ and CD8^+^ T cells, of which the downregulation promotes T‐cell IFN‐γ‐mediated antitumour immunity and inhibits tumour growth. In addition to LC, we also noticed that Tim3‐associated immune reactions occurred in acute pneumonia and stable pneumonia. Our results also indicate that Eomes‐driven differentiation and function of NK cells and Eomes‐induced Tex are obviously active in acute pneumonia, while less active in COPD and AECOPD. The age of patients with COPD and AECOPD may be one of the influencing factors for such variations between lung diseases. These results indicate that molecule mechanisms in the immune modulation in various lung diseases are more complex than we expected.

Cell therapeutics for diseases has been studied by researchers, and treatment for acute respiratory distress syndrome (ARDS) with bone marrow‐derived mesenchymal stromal cells had been effective in preclinical models or clinical trials.^66^, ^67^ As a new type of interstitial cells, TCs had been demonstrated to improve lung injury in ARDS models and alleviate LPS‐induced vascular endothelial cell proliferation inhibition.^68^ However, the effect of TCs on immune cells and the mechanisms had been unclear. Recently, studies summarized paracrine effects of MSCs in reducing lung injury and enhancing lung repair in ARDS and sepsis.^69^ Our current studies using contact or noncontact co‐culture system elaborated effects of TCs or its paracrine on the expression levels and distribution of immune markers in PBIC of patients with acute pneumonia. The mechanisms involved in the effect of TCs on T cells, granulocytes or NK cells should be discussed in depth.

In conclusion, the present study identified differences in the proportions and distribution of PBIC clusters and functions among lung diseases at different molecular levels of proteomics, transcriptomics and phenomics, at various stages and natures of the disease, and at multiorientations of understanding. We noticed that the peripheral immune states, responses, differentiations and regulations of lung diseases vary between acute and stable conditions, between cancer and noncancer stages, between infection and inflammation, and between allergy and nonallergy. Moreover, we found that the exhausted CD4^+^ T subsets exist in acute pneumonia and stable pneumonia. The difference of biological processes and pathways of PBICs by single‐cell sequencing and RNAseq varies among diseases. The interstitial cells could alter the differentiation and development of the immune cell types and immune function. Thus, new clusters, subsets and functions of circulating immune cells can be important sources to understand molecular mechanisms, identify disease‐specific biomarkers and develop new target therapies.

## CONFLICT OF INTEREST

The authors declare that there is no conflict of interest.

## Supporting information


**Supporting Figure S1** Distribution of immune molecules in healthy volunteers and patients with lung disease

**Supporting Figure S2** T‐cell immune patterns across healthy volunteers and patients with lung diseases. (A) Boxplots showing the frequencies of the indicated T‐cell clusters that have alteration in the PBMC of healthy controls, acute pneumonia and stable pneumonia patients. (B) Boxplots showing the frequencies of the indicated T‐cell clusters that have alteration in the PBMC of healthy controls and patients with LC. (C) Boxplots showing the frequencies of the indicated T‐cell clusters that have alteration in the PBMC of healthy controls, AECOPD and COPD patients. (D) Boxplots showing the frequencies of the indicated T‐cell clusters that have alteration in the PBMC of healthy controls, acute asthma and stable asthma patients

**Supporting Figure S3** T‐cell immune patterns across healthy volunteers and patients with acute pneumonia, stable pneumonia, acute asthma, stable asthma, COPD, AECOPD and LC

**Supporting Figure S4** Boxplots showing the frequencies of the indicated immune molecules that have alteration in cluster 03 of T cells

**Supporting Figure S5** Identification of primary TCs. Vimentin (green), PDGFRα (purple), FOXL1 (red) were used for TCs identification and the nucleus was stained by DAPI (blue)

**Supporting Table S1** Stages of lung cancer patients
**Supporting Table S2** Antibodies information
