# Supplementary material for: A cellular census of human peripheral immune cells identifies novel cell states in lung diseases
Source: Clin Transl Med. 2021 Nov 24;11(11):e579. doi: 10.1002/ctm2.579 (PMC8611783; doi:10.1002/ctm2.579)
Supplement: Supplementary file 6 — Supporting Table S1 Stages of lung cancer patients Supporting Table S2 Antibodies information [file CTM2-11-e579-s003.docx]

**Supplemental Table1.** **Stages of lung cancer patients.**

|  | LC Patient 1 | LC Patient 2 | LC Patient 3 | LC Patient 4 | LC Patient 5 |
| --- | --- | --- | --- | --- | --- |
| Prognosis | Squamous cell carcinoma | Squamous cell carcinoma | Squamous cell carcinoma | Adenocarcinoma | Adenocarcinoma |
| Clinical stage | ⅢC | IVA | IVB | ⅢB | IVB |
| Primary Tumor | 4 | 2b | 2a | 1c | 1 |
| Regional Lymph Nodes | 2 | 3 | 3 | 3 | 2 |
| Distant Metastasis | 0 | 1a | 1c | 0 | 1c |
| Karnofsky | 0 | 1 | 1 | 0 | 0 |

**Supplemental Table 2. Anttibodies information.**

| Label | Marker | Clone | Manufactor |
| --- | --- | --- | --- |
| 89Y | CD45 | HI30 | BioLegend |
| 115ln | CD3 | UCHT1 | BIO X CELL |
| 139La | CD47 | CC2C6 | BioLegend |
| 141Pr | CD56 | NCAM16.2 | BD Bioscience |
| 142Nd | CD19 | HIB19 | BioLegend |
| 142Nd | TCRgd | 5A6.E9 | Plttech |
| 143Nd | CD27 | O323 | BioLegend |
| 144Nd | CD14 | M5E2 | BioLegend |
| 145Nd | CD270 | 122 | BioLegend |
| 146Nd | CD123 | 6H6 | BioLegend |
| 147Sm | CD66b | G10F5 | BioLegend |
| 148Nd | TIGIT | A15153G | BioLegend |
| 149Sm | CD25 | 24212 | R&D Systems |
| 150Nd | CD223_Lag3 | 874501 | R&D Systems |
| 151Eu | CD38 | HIT2 | BioLegend |
| 152Sm | CD39 | A1 | BioLegend |
| 153Eu | CD274_PD_L1 | 29E.2A3 | BioLegend |
| 154Sm | TCRab | IP26 | BioLegend |
| 155Gd | CD45RA | HI100 | BioLegend |
| 156Gd | CD59 | P282（HI9) | BioLegend |
| 157Gd | CD1c | L161 | BioLegend |
| 158Gd | CD197_CCR7 | G043H7 | BioLegend |
| 159Tb | CD11c | BU15 | BioLegend |
| 160Gd | CD28 | CD28.2 | BioLegend |
| 161dy | CD152_CTLA_4 | 14D3 | eBioscience |
| 162Dy | FoxP3 | PCH101 | eBioscience |
| 163Dy | CD137_4_1BB | 4B4-1 | BioLegend |
| 164Dy | RORγ | 600214 | R&D Systems |
| 165Ho | CD366_Tim3 | F38-2E2 | BioLegend |
| 166Er | CD134_OX40 | BER-ACT35 | BioLegend |
| 167Er | CD45RO | UCHL1 | BioLegend |
| 168Er | Tbet | 4B10 | BioLegend |
| 169Tm | CD154_CD40L | 24-31 | BioLegend |
| 170Er | CD127_IL_7Ra | A019D5 | BioLegend |
| 171Yb | GATA3 | TWAJ | eBioscience |
| 172Yb | Eomes | 644730 | R&D Systems |
| 173Yb | Granzyme B | GB11 | Fluidigm |
| 174Yb | CD279_PD-1 | EH12.2H7 | BioLegend |
| 175Lu | CD16 | 3G8 | BioLegend |
| 176Yb | HLA_DR | L243 | BioLegend |
| 197gd | CD4 | RPA-T4 | BioLegend |
| 198pt | CD8a | RPA-T8 | BioLegend |
| 209Bi | CD11b | M1/70 | BioLegend |
